# Magnetic resonance imaging for optimized implantation and long-term monitoring of patients receiving a left atrial appendage occluder

**DOI:** 10.1186/1532-429X-13-S1-P255

**Published:** 2011-02-02

**Authors:** Anil-Martin Sinha, Harald Rittger, Christian Mahnkopf, Stefan Holzmannl, Manfred Duecker, Abed Sallam, Guido Ritscher, Johannes Brachmann

**Affiliations:** 1 Klinikum Coburg, Coburg, Germany

## Introduction

Patients with atrial fibrillation (AF) often suffer from blood clots originating from the left atrial appendage (LAA). As these clots are the main cause of central and peripheral embolism, patients receive oral anticoagulation therapy. For those patients who cannot be treated effectively with oral anticoagulants, percutaneous transcatheter LAA occlusion systems have been invented as a promising alternative.

## Purpose

To test the feasibility of cardiac magnetic resonance imaging (CMR) for device size selection and for monitoring LAA occlusion in AF patients.

## Methods

Patients scheduled for LAA occlusion received CMR before and after device deployment. Left atrial angiography, and 3D navigator and delayed enhancement sequences were applied. The size of the left atrium (LA), LAA, and position and size of the occluder were measured.

## Results

**Figure 1:**
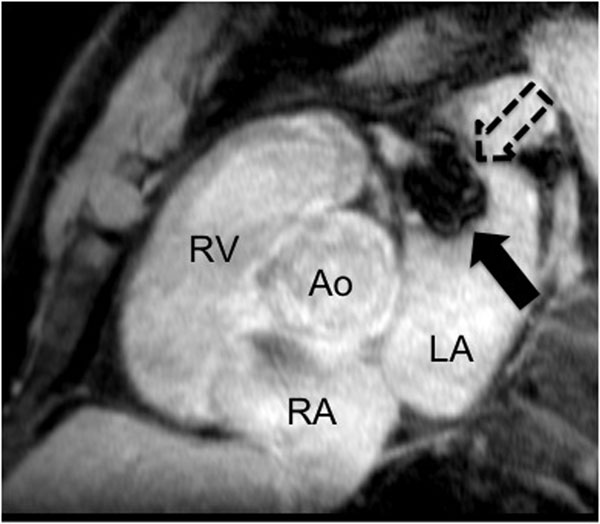


10 patients (7 male, age 71±10 years) received a LAA occluder system (Amplatzer, AGA Medical Corp, Golden Valley/ MN, USA). 4/10 patients suffered from paroxymal, 6/10 from permanent AF, 6/10 from hypertension, and 4/10 from coronary artery disease with 2 having a prior myocardial infarction. 9/10 had a history of at least one stroke, 1/10 of TIA. CMR (Siemens Espree 1.5T, Germany) showed an ejection fraction of 58.3±5.2%, LA diameter of 58.1±17.2mm, and LAA diameter of 25.7±4.5mm before device implantation. Manufacturing occluder diameters were 25.1±4.7mm, resulting in a difference of 1.6±0.3mm as compared to measured LAA. After a follow-up period of 24±4 weeks, there was no device dislocation or thrombotic formation detected by CMR. LA diameter was 54.1±7.6 mm, LAA diameter 27.9±3.2mm, and occluder diameter 25.3±4.9mm. 9/10 occluders were leakproof, 1/10 was a little untight, showing a small contrast agent rim around the occluder. Figure 1 shows an CMR example after LAA occluder implantation (Ao=aorta, RA=right atrium, RV=right ventricle, full arrow=LAA occluder).

## Conclusion

CMR allows visualization of LAA and selection of the optimal LAA occluder size. CMR is feasible in patients after LAA occluder implantation, and seems to be safe for evaluation of device position, size, and tightness. Therefore, CMR seems to be a valuable visualization tool for LAA occluder implantation, and should be routinely used for long-term monitoring of device deployment success.

